# Error in Funding/Support Statement

**DOI:** 10.1001/jamanetworkopen.2023.7341

**Published:** 2023-03-21

In the Original Investigation titled “Assessment of Alcohol Exposure From Alcohol-Based Disinfectants Among Premature Infants in Neonatal Incubators in Japan,”^1^ published February 24, 2023, there was an error in the Funding/Support statement in the end matter. The number given as JP 21lm0203010 should have been JP 22ym0126803. This article has been corrected.^1^
